# Effects of Time-Restricted Fasting–Nicotinamide Mononucleotide Combination on Exercise Capacity via Mitochondrial Activation and Gut Microbiota Modulation

**DOI:** 10.3390/nu17091467

**Published:** 2025-04-26

**Authors:** Jian Shi, Tingting Zhuang, Weiye Li, Xueping Wu, Junming Wang, Ruiying Lyu, Jingxin Chen, Chunhong Liu

**Affiliations:** 1 College of Food Science, South China Agricultural University, Guangzhou 510642, China; clinicalshijian@foxmail.com (J.S.); 18302487939@163.com (W.L.); xp158165@163.com (X.W.); 20223141071@stu.scau.edu.cn (J.W.); 20223185044@stu.scau.edu.cn (R.L.); chenjingxin4829@163.com (J.C.); 2 Guangdong Provincial Key Laboratory of Food Quality and Safety, Guangzhou 510642, China; 3 College of Agricultural Engineering, Guangdong Meizhou Vocational and Technical College, Meizhou 514028, China; keahu3@163.com

**Keywords:** time-restricted fasting, nicotinamide mononucleotide, mitochondria function, athletic performance

## Abstract

Background/Objectives: Athletic performance matters for athletes and fitness enthusiasts. Scientific dietary intervention may boost athletic performance alongside training. Intermittent fasting, like time-restricted fasting (TF), may enhance metabolic health. NAD^+^ supplement nicotinamide mononucleotide (NMN) improves mitochondrial activity. Both potentially boost athletic performance. However, whether TF combined with NMN treatment can further enhance athletic ability is unclear. Methods: Healthy Kunming mice were utilized to test the effects of NMN and TF on the athletic performance of mice. To simulate the in vivo state and further verify the role of TF and NMN, low glucose combined with NMN was used to intervene in C2C12 cells. The exercise capacity of mice was evaluated through motor behavior experiments. At the same time, blood gas analysis and kit tests were used to assess oxygen uptake capacity and post-exercise oxidative stress levels. Muscle development and mitochondrial function were examined through gene expression, protein analysis, and enzyme activity tests, and the distribution of intestinal microbiota and short-chain fatty acid content were also analyzed. Results: The results show that TF combined with NMN improved mitochondrial dynamics and biosynthesis, mitochondrial respiratory function, and oxidative metabolism. Then, the intervention enhanced mice’s endurance, limb strength, motor coordination, and balance and reduced oxidative damage after exercise. Moreover, TF combined with NMN significantly increased the gut microbiota diversity and upregulated *Ruminococcus*, *Roseburia*, and *Akkermansia* in intestinal bacteria and short-chain fatty acids, which are associated with athletic performance. Conclusion: TF combined with NMN enhanced mitochondrial function, improved energy metabolism, modulated the gut microbiota and short-chain fatty acids, and affected muscle fiber transformation, ultimately leading to an overall improvement in exercise performance. These findings provide a theoretical framework for expanding the application of NMN and TF in kinesiology.

## 1. Introduction

Mitochondria possess the remarkable capacity to adapt to metabolic demands at subcellular, cellular, and tissue-specific levels [1,2]. During exercise, skeletal muscle mitochondria demonstrate enhanced ATP-generating capacity to meet increased energy requirements [3]. As the primary site of ATP production—accounting for approximately 90% of intracellular ATP—mitochondria support homeostasis primarily through oxidative phosphorylation (OXPHOS). Additionally, they modulate various cellular processes, including gluconeogenesis, ion homeostasis, and apoptosis, via ATP production and redox-sensitive signaling pathways [4]. These metabolic adaptations are often accompanied by increased expression of genes involved in muscle structure and mitochondrial oxidative metabolism [5,6]. Thus, strategies that enhance mitochondrial function are of interest for improving athletic performance through more efficient energy metabolism.

Nutritional interventions such as time-restricted fasting (TF) have emerged as promising tools for optimizing metabolic health and exercise performance [7,8]. TF, a well-characterized form of intermittent fasting, restricts food intake to a daily window of 6–12 h and has demonstrated benefits in reducing energy imbalance, improving body composition, and preserving physical performance [9,10,11,12]. Notably, TF can influence mitochondrial pathways such as OXPHOS and the tricarboxylic acid (TCA) cycle, thereby enhancing oxygen utilization and ATP production—critical determinants of endurance and power output [13,14,15,16].

Nicotinamide adenine dinucleotide (NAD^+^) is a key cofactor in cellular energy metabolism, acting as an essential substrate in the TCA cycle and OXPHOS through its redox couple with NADH [17,18]. Nicotinamide mononucleotide (NMN), a key precursor in NAD^+^ biosynthesis, is metabolized via NMNAT1 (nuclear) and NMNAT3 (mitochondrial), supporting intracellular NAD^+^ production [19]. Supplementation with NMN has been shown to improve mitochondrial respiration and enhance OXPHOS capacity, even after short-term administration [20,21]. Furthermore, NMN has been associated with enhanced physical performance, as demonstrated through cardiopulmonary function and skeletal muscle endurance markers [22].

The widespread use of dietary supplements in sports underscores their perceived value in enhancing performance, optimizing body composition, and accelerating recovery [23]. Skeletal muscle, as the primary effector of movement and metabolic regulation, is central to these enhancements. While both TF and NMN independently promote mitochondrial function and metabolic health, their potential synergistic effects on exercise performance have yet to be fully elucidated.

In this study, the combined effects of TF and NMN at various dosages on exercise performance were investigated in Kunming mice, alongside in vitro studies using C2C12 cells under low-glucose conditions to mimic fasting states. Mitochondrial respiratory function, respiratory chain complex enzyme activity, and gut microbiota composition were evaluated to explore mechanisms underlying these effects. Our findings provide novel insights into how the combined intervention of TF and NMN may enhance metabolic flexibility and exercise capacity, offering practical implications for athletes and performance optimization strategies.

## 2. Materials and Methods

### 2.1. Animal Treatment

Sixty-five male Kunming mice (5 weeks old; 27.55 ± 1.19 g prior to the adaptation period) were housed under controlled environmental conditions (22 ± 0.5 °C, 50–60% relative humidity, 12 h light/dark cycle) and provided a standard diet for a 7-day acclimation period. The composition of the standard diet is detailed in Table S1. Following acclimation, the mice were randomly assigned to one of five groups based on feeding regimen and NMN gavage dosage: (1) ad libitum diet + 0 mg/kg NMN (control), (2) time-restricted fasting (TF) + 0 mg/kg NMN (TF), (3) TF + 125 mg/kg NMN (TFNL), (4) TF + 250 mg/kg NMN (TFNM), and (5) TF + 500 mg/kg NMN (TFNH). NMN or the vehicle was administered daily via oral gavage at 08:00, prior to recording body weight and feed intake. The intervention lasted for six weeks, as illustrated in the schematic diagram (Figure S1). Body composition analysis and behavioral assessments were performed at the end of the intervention period. The body composition analysis quantified fat, lean, and water mass using a MesoQMR awake small animal body composition analyzer (QMR23-060H, Niumag, Suzhou, China). Then, the mice were euthanized with sodium pentobarbital. The cardiac arterial blood (1 mL) was quickly collected and injected into the sample slot of a blood gas analyzer (Model XQ-101, Wondfo Instruments, Guangzhou, China) for blood gas analysis after sufficient shaking. Gastrocnemius muscles and livers were immediately harvested, rapidly frozen in liquid nitrogen, and stored at −80 °C following cervical dislocation. Each individual mouse was considered an independent experimental unit. All animal procedures were approved by the Animal Care and Use Committee of South China Agricultural University (Approval No. 2024B031). To minimize unnecessary animal use, subsequent validation experiments were conducted using cell-based models, eliminating the need for additional animal studies.

### 2.2. Cell Treatment

C2C12 cells were obtained from Warner Biotechnology (Wuhan, China) and cultured in high-glucose DMEM (4500 mg/L) supplemented with 10% fetal bovine serum (FBS) in a 37 °C incubator with 5% CO_2_. Once the cells reached approximately 80% confluence, the growth medium was replaced with a differentiation medium containing 2% horse serum to induce myotube formation. After six days, regularly aligned mature myotubes were observed under a microscope, indicating successful differentiation.

On day 5 of differentiation, C2C12 cells were treated with either 500 μM nicotinamide mononucleotide (NMN), a low-glucose (LG; 1000 mg/L) medium, or a combination of both for 24 h. Additional groups were treated with 125 μM, 250 μM, or 500 μM NMN in combination with LG to evaluate dose-dependent effects.

### 2.3. Exercise Capacity Testing

#### 2.3.1. Treadmill Exhaustion Test

The exercise protocol was adapted from a previous study [24]. Mice were subjected to treadmill running using a KM-PT/8 treadmill (KEW Basis, Nanjing, China) equipped with eight angle-adjustable lanes. Following three days of acclimation, an exhaustion test was conducted on the subsequent day. The treadmill speed was initially set to 10 m/min and increased by 5 m/min every 5 min. The incline angle was kept at 15° throughout the test. Exhaustion was defined as the inability to resume running after a 5-s exposure to the electric grid, which delivered a 0.2 mA stimulus according to animal welfare guidelines.

The total work output (J) was calculated using the following formula: Total work done (J) = Body weight (kg) × Exhaustion distance (m) × sin (θ) × 9.8 (J/kg × m), where θ represents the angle between the treadmill surface and the ground.

#### 2.3.2. Forced Swimming Test

The exercise training protocol was adapted from a previous study [25]. Mice were placed individually in a plastic container (25 cm in height, 20 cm in diameter) filled with tap water to a depth of 10–11 cm, maintained at 30 ± 1 °C. A load equivalent to 10% of each mouse’s body weight was attached during training.

#### 2.3.3. Wire Hang Test

For the wire hang test, mice were positioned on a wire mesh gently inverted and slightly shaken to encourage them to grasp the wire. The latency to fall was recorded as an indicator of muscle endurance.

#### 2.3.4. Climbing-Pole Test

Motor strength and coordination were assessed using the pole test, as previously described [26]. Mice were gently placed in a head-down position on top of a vertical pole (~50 cm in length, ~1 cm in diameter) wrapped in gauze for traction. The time taken for the mouse to descend from the top to the base of the pole was recorded as the climbing time.

#### 2.3.5. Rotarod Test

The rotarod test was employed to evaluate motor coordination and balance, as previously described [27]. Mice were placed on a rotating rod (4 cm in diameter) set at a constant speed of 30 rpm, and the latency to fall was recorded as the primary outcome. All mice underwent the assessment without prior training to eliminate adaptation effects.

#### 2.3.6. Strength Test

As previously described, grip strength was evaluated using a standardized weight-lifting test to assess muscular strength in mice [28]. Each mouse was gently held by the mid-tail and allowed to grasp a gauze-wrapped weight. The test included four incremental weight levels—20 g, 40 g, 60 g, and 80 g—corresponding to point values of 1, 2, 3, and 4, respectively. Mice progressed to the next level upon successfully maintaining their grip for a continuous duration of 3 s.

The scoring system comprised two components.

Base score: calculated by multiplying the point value of each fully completed level (i.e., prior to the final level attempted) by 3 s and then summing these values.

Highest score: determined by multiplying the point value of the final weight level attempted by the actual number of seconds the mouse maintained its grip.

The total score was the sum of the base score and the highest score.

### 2.4. Histology Staining, Immunohistochemistry, and Immunofluorescence

The gastrocnemius muscles were fixed in 4% formaldehyde to evaluate muscle fiber morphology. After routine dewaxing and rehydration, 4 μm paraffin-embedded sections were subjected to periodic acid–Schiff (PAS), Masson’s trichrome, immunohistochemical (IHC), and immunofluorescence staining. Stained sections were examined using a light microscope (Leica, Wetzlar, Germany). Histological alterations in the gastrocnemius were evaluated in at least three randomly selected fields per sample. Details of the primary antibodies used are provided in Table S2.

### 2.5. Measurement of Oxidative Damage and Antioxidant Markers

The serum levels of 8-iso-prostaglandin F2α (8-iso-PGF2α), 8-hydroxy-2′-deoxyguanosine (8-OHdG) and protein carbonyls (PCO) were measured using corresponding commercial assay kits following the treadmill exhaustion test. According to the manufacturer’s protocols, the gastrocnemius muscles were analyzed for total superoxide dismutase (SOD) activity and the levels of malondialdehyde (MDA), reduced glutathione (GSH), and oxidized glutathione (GSSG).

### 2.6. Detection of Energy Metabolism-Related Indicators

LDH and PDH play a key role in energy metabolism, and NMNAT is a rate-limiting enzyme in NAD^+^ metabolism. Therefore, mature commercial kits were used to detect the enzyme activities of LDH, PDH, and NMNAT. ATP content is a direct reflection of the level of energy metabolism. In this study, ATP in gastrocnemii was detected using an ATP chemiluminescence assay kit. Briefly, samples were lysed with RIPA lysis buffer and centrifuged at 12,000× *g* for 5 min at 4 °C, and the supernatant was collected for ATP measurement. OXPHOS is the main way to produce ATP and depends on the enzyme activity of the mitochondrial respiratory chain complex enzymes (Cx). As described previously, the Cx I, II, III, and IV activity were evaluated via ultraviolet spectrophotometry [29].

### 2.7. Determination of Mitochondrial Respiratory Function

The mitochondrial oxygen consumption rate (OCR) was measured using an Oxygraph-2k instrument (Oroboros, Innsbruck, Austria). Equal amounts of gastrocnemius homogenate were introduced into the respiration chamber containing the respiratory reaction buffer. OCR was subsequently recorded in response to mitochondrial substrates (succinate, pyruvate, and malate), ADP, carbonyl cyanide m-chlorophenylhydrazone (CCCP), rotenone, and antimycin A.

### 2.8. Detection of Mitochondrial DNA Copy Number

The mitochondrial DNA (mtDNA) copy number was quantified using qPCR. DNA was extracted from gastrocnemius tissue using SDS, proteinase K, and phenol/chloroform. Primer sequences, as detailed in Table S3, were selected from previous studies: 36B4 for the nuclear genome and Cytb for the mitochondrial genome [30].

### 2.9. Detection of Gene Transcription

Total RNA from the gastrocnemius was extracted using a TRIzol reagent (TaKaRa, Kusatsu, Japan). Reverse transcription was performed using reverse transcriptase, and SYBR Green qPCR Premix (Xinkailai, Guangzhou, China) was used for quantitative PCR analysis, conducted on a QuantStudio 3 real-time PCR system (Thermo Fisher, Waltham, MA, USA). Primer sequences are provided in Table S3. GAPDH was used as the reference gene, and relative gene expression levels were calculated using the 2−ΔΔCt method.

### 2.10. Western Blot Analysis

For Western blot analysis, tissue and cell lysates were prepared using a RIPA lysis buffer (Beyotime, Shanghai, China). Equal amounts of total protein, quantified using the BCA assay, were diluted in 5 × SDS-PAGE loading buffer and boiled for 5 min. Proteins were separated via SDS-PAGE and transferred to PVDF membranes following standard protocols. The membranes were blocked with TBST buffer containing 5% skim milk powder or BSA and incubated with diluted primary antibodies for 16 h. Afterward, the membranes were probed with the appropriate HRP-conjugated secondary antibodies. Band intensities were normalized to GAPDH or Actin as loading controls, and protein levels were quantified using the ImageJ software (1.51). Information on the primary antibodies used is provided in Table S2.

### 2.11. Analysis of Gut Microbiota and Short-Chain Fatty Acids (SCFAs)

Microbial DNA from mouse feces was quantified with a Qubit kit (Invitrogen, Waltham, USA), and PCR products were sequenced on an Illumina NovaSeq 6000 instrument. Statistical analysis was performed in R (version 4.0.0). The concentrations of acetic acid, propionic acid, isobutyric acid, butyric acid, isovaleric acid, and valeric acid in the cecal contents were quantified using methods described in previous studies [31].

### 2.12. Statistical Analysis

The data were presented as means ± SD. Statistical analysis using SPSS 22.0 and graphing using GraphPad Prism 9.2.0 were performed. If equal variances were assumed, differences were assessed via a one-way ANOVA, followed by Tukey’s HSD post hoc test. A significance level of *p* < 0.05 was considered statistically significant.

## 3. Results

### 3.1. Effect of TF and NMN Supplementation on Physiological Index of Mice

**Figure 1:**
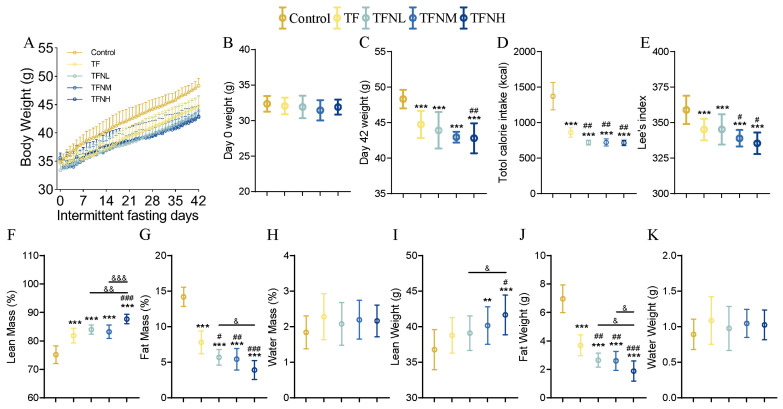
Physiological indices of mice. (**A**) The body weights of mice in each group from day 0 to day 42 of the experiment trends. (**B**) Body weight on day 0. (**C**) Body weight on day 42. (**D**) Total calorie intake during the experiment. (**E**) Lee’s index. (**F**) Lean mass (%). (**G**) Fat mass (%). (**H**) Water mass (%). (**I**) Lean weight (g). (**J**) Fat weight (g). (**K**) Water weight (g). Data were presented as means ± SDs. * represents the difference from the control group, # represents the difference from the TF group, and & represents the difference between the combined intervention groups. #, &: *p* < 0.05, **, ##, &&: *p* < 0.01, and ***, ###, &&&: *p* < 0.001.

Daily weight changes were monitored to evaluate the effects of TF and TF supplementation with NMN on body weight and condition. Body composition analyses were conducted at the end of the experimental period. Figure 1A shows the variation in body weight for each group throughout the study. During the first two weeks, body weight fluctuations were observed in all groups, after which the weight stabilized, remaining consistently lower than that of the control group for most of the study period. After 42 days of intervention with TF or TF combined with NMN (TFN), the mice exhibited a significant reduction in body weight compared to the control group, with the TFNH group showing a notably substantial decrease than the TF group (Figure 1B,C). The total caloric intake in the TF group was significantly lower than in the control group throughout the trial and further decreased upon NMN supplementation (Figure 1D). At the end of the intervention, the mice’s body length and Lee’s index were measured and calculated (Figure 1E). TF and TFN led to significantly leaner mice, with the TFNH group being considerably leaner than the TF and TFNL groups. A body mass analysis (Figure 1F–H) revealed that TF significantly increased lean mass (%), which was further elevated with NMN administration. Notably, the TFNH group exhibited substantially higher lean mass than the TFNL group. Conversely, fat mass (%) followed an inverse pattern, with the TF group showing a marked reduction compared to the control and TFN further reducing fat mass dose-dependently. The mice also gained lean weight (g) while losing fat weight (g) (Figure 1I–K).

### 3.2. Effect of TF and TFN on Exercise Performance and Relative Indicators of Mice

**Figure 2:**
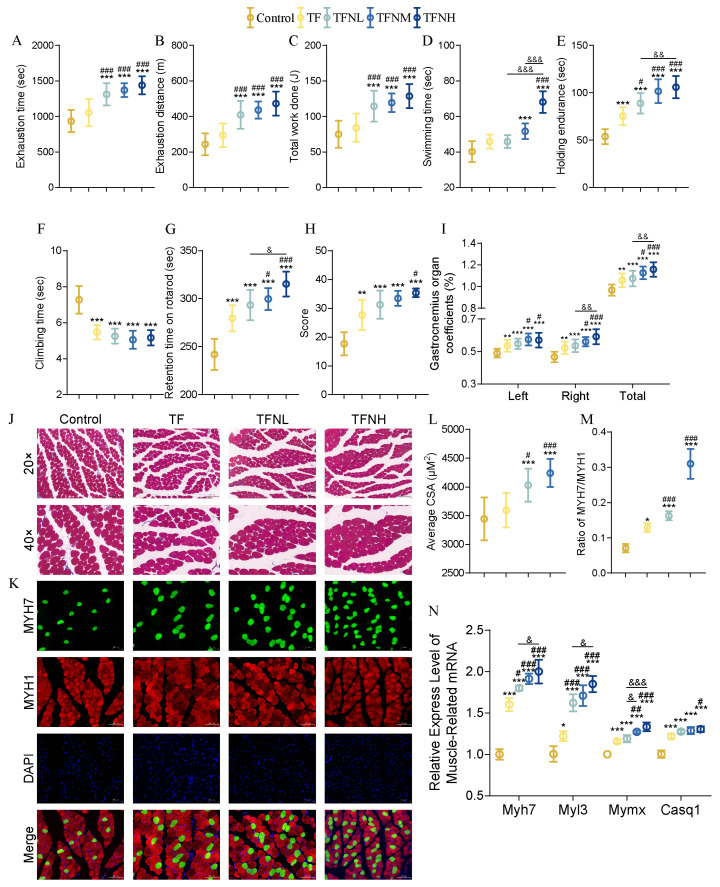
Athletic performance and muscle fiber morphology. (**A**) Average exhaustion time. (**B**) Average exhaustion distance. (**C**) Average total work done in the running. (**D**) Average forced swimming time. (**E**) Wire-hang holding time. (**F**) Climbing-bar descent time. (**G**) Retention time on rotarod. (**H**) Strength score. (**I**) Gastrocnemius organ coefficients. (**J**) Tissue section Masson staining of gastrocnemius. (**K**) Immunofluorescent dual staining. (**L**) Muscle fiber cross-sectional area. (**M**) The ratio of fast and slow muscle fibers. (**N**) Relative expression of muscle-related genes (Myh7, Myl3, Mymx, and Casq1). Data were presented as means ± SDs. * represents the difference from the control group, # represents the difference from the TF group, and & represents the difference between the combined intervention groups. *, #, &: *p* < 0.05, **, ##, &&: *p* < 0.01, and ***, ###, &&&: *p* < 0.001.

The athletic ability of the mice was assessed through various behavioral tests, including the treadmill exhaustion test, forced swimming test, wire hang test, climbing-pole test, rotarod test, and strength test. Given the relationship between muscle and athletic performance, the gastrocnemius muscle was focused on, calculating its muscle coefficient, observing its fiber morphology, measuring the cross-sectional area (CSA) of muscle fibers, and evaluating the distribution and proportion of different muscle fiber types. Additionally, the expression of genes related to muscle development was examined to explore the effects of time-restricted fasting (TF) and TF combined with nicotinamide mononucleotide (NMN) on athletic performance and possible influencing factors at the muscle level. TF significantly prolonged the exhaustion time in the treadmill exhaustion test (Figure 2A–C). Although there were no significant differences in the exhaustion distance and total work done compared to the control group, TFN significantly improved all three parameters, outperforming both the control and TF groups in a dose-dependent manner. The TFNH group achieved 1.94 times the exhaustion distance of the control group. In the forced swimming test (Figure 2D), both TF and TFN substantially increased the swimming time, with the TFNM and TFNH groups showing even more significant improvements over the TF group. The TFNH group swam significantly longer than the TFNL group. In the wire hang test (Figure 2E), both TF and TFN enhanced endurance, improving performance in line with the NMN dosage. The climbing-pole test revealed significantly reduced climbing times in the TF and TFN groups (Figure 2F). In the rotarod test, both TF and TFN showed marked benefits, with retention time positively correlated with the NMN dose (Figure 2G). Strength scores in the strength test were significantly higher in all experimental groups compared to the control group, with the TFNH group exhibiting a notably more excellent score than the TF group (Figure 2H). To assess muscle status, the gastrocnemius muscle coefficient was calculated (Figure 2I). TF and TFN significantly increased this coefficient, with the TFNM and TFNH groups showing further enhancement compared to TF. The correct and total gastrocnemius coefficients in the TFNH group were considerably higher than those in the TFNL group. Muscle fiber morphology was further assessed (Figure 2J), alongside immunofluorescence double staining of MYH7 and MYH1 to identify and quantify fast- and slow-twitch muscle fibers (Figure 2K). TFN significantly increased the CSA of gastrocnemius muscle fibers (Figure 2L) and increased the proportion of slow-twitch muscle fibers in a dose-dependent manner (Figure 2M). Regarding muscle-related genes, TF and TFN significantly elevated the expression of Myh7, Myl3, Mymx, and Casq1, with these increases corresponding to NMN dosage (Figure 2N).

### 3.3. Effect of TF and TFN on Oxidation and Antioxidant Levels of Mice

**Figure 3:**
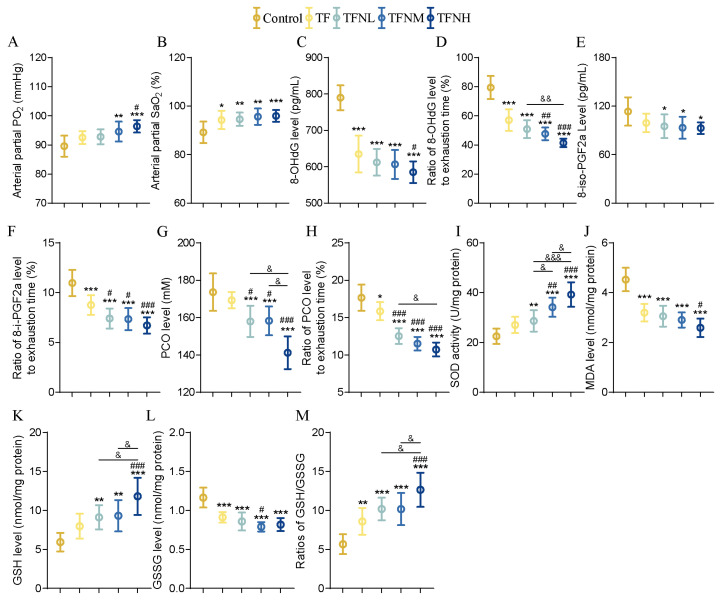
Oxidation and antioxidant levels of mice. (**A**) PO_2_ in mouse arterial blood. (**B**) SaO_2_ in mouse arterial blood. (**C**) 8-OHdG content after exercise. (**D**) The ratio of 8-OHdG content to exhaustion time. (**E**) 8-i-PGF2a content after exercise. (**F**) The ratio of 8-i-PGF2a content to exhaustion time. (**G**) PCO content after exercise. (**H**) The ratio of PCO content to exhaustion time. (**I**) SOD activity of gastrocnemius. (**J**) MDA concentration of gastrocnemius. (**K**–**M**) GSH and GSSG levels, and the ratios of GSH/GSSG. Data were presented as means ± SDs. * represents the difference from the control group, # represents the difference from the TF group, and & represents the difference between the combined intervention groups. *, #, &: *p* < 0.05, **, ##, &&: *p* < 0.01, and ***, ###, &&&: *p* < 0.001.

Aerobic exercise is directly related to the body’s oxygen uptake capacity. Additionally, intense exercise generates a large number of free radicals in muscles. These free radicals can damage biological macromolecules such as cell membranes, proteins, and nucleic acids, leading to oxidative stress. Therefore, blood gas analysis and oxidative stress-related indicators were employed to assess mice’s exercise capacity indirectly. Following treatment with TF or TFN, the partial pressure of oxygen (PO_2_) showed a significant increase, with the TFNH group demonstrating a notably higher level than the TF and TFNL groups (Figure 3A). Furthermore, TF and TFN significantly enhanced arterial oxygen saturation (SaO_2_) in mice compared to the control group (Figure 3B). Figure 3C shows that TF and TFN significantly reduced 8-OHdG levels, with no substantial difference between the TF and TFN groups. TFNH further decreased 8-iso-PGF2a levels compared to TF (Figure 3E). The effects of TF and TFN on PCO were more pronounced (Figure 3G), with blood PCO levels in the TFN groups being significantly lower than those in the TF group and the effect of NMN showing a dose-dependent relationship. The ratios of 8-OHdG, 8-iso-PGF2a, and PCO to exhaustion times, reflecting antioxidant damage, indicated that TFN further reduced both 8-iso-PGF2a and PCO, with dose-dependent effects observed for both markers (Figure 3D,F,H). On the other hand, TF and TFN significantly increased SOD levels in the gastrocnemius, with increases correlating positively with NMN concentrations (Figure 3I). TF also significantly reduced gastrocnemius MDA levels, and TFNH showed a considerable reduction influenced by both TF and TFNL (Figure 3J). Additionally, the levels of GSH and GSSG, as well as their ratio, underwent substantial changes following TF and TFN interventions (Figure 3K–M).

### 3.4. Effect of TF and TFN on Energy Metabolism of Mice

**Figure 4:**
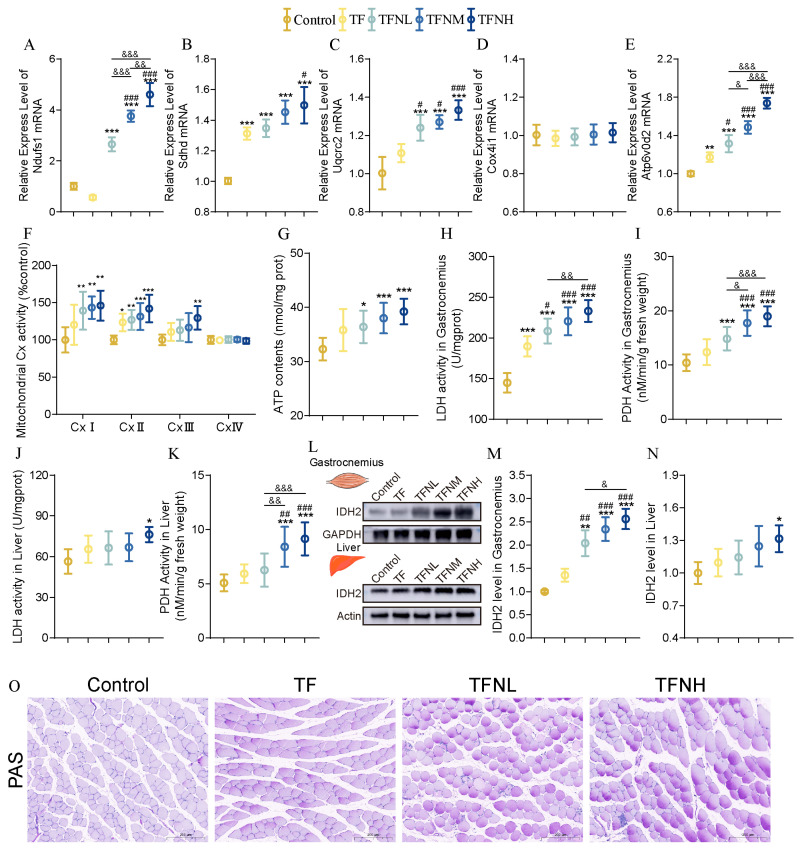
Energy metabolism-related indicators. (**A**–**E**) Relative expression of genes for OXPHOS subunits (Ndufs1, Sdhd, Uqcrc2, Cox4i1, Atp6v0d2). (**F**) Activities of the mitochondrial respiratory chain complex enzyme. (**G**) ATP content of gastrocnemius. (**H**) gastrocnemius LDH enzyme activity. (**I**) gastrocnemius PDH enzyme activity. (**J**) hepatic LDH enzyme activity. (**K**) hepatic PDH enzyme activity. (**L**–**N**) Relative expression of IDH2 in gastrocnemius and the liver. (**O**) Gastrocnemius muscle glycogen PAS staining. Data were presented as means ± SDs. * represents the difference from the control group, # represents the difference from the TF group, and & represents the difference between the combined intervention groups. *, #, &: *p* < 0.05, **, ##, &&: *p* < 0.01, and ***, ###, &&&: *p* < 0.001.

Energy metabolism was evaluated to explore the biological mechanism of improved athletic performance caused by TF and TFN evaluated in gastrocnemius. For the gene expression levels of OXPHOS subunits and the activities of mitochondrial complexes (Figure 4A–F), TF markedly decreased Ndufs1 levels, although it did not substantially alter CxI activity. Both gene expression and enzyme activity rose considerably when paired with NMN. TF markedly enhanced the expression of Sdhd, Uqcrc2, Atp6v0d2, and the activity of CxII but not CxIII. TFN further substantially elevated gene expression and activity in conjunction with a dose effect. Furthermore, neither TF nor TFN substantially impacted Cox4i1 and CxIV. The TF and TFN groups’ ATP generations were increased in both the TF and TFN groups, with the TFNH group having the highest ATP content, which was significantly higher than the TF and TFNL groups (Figure 4G). Alongside OXPHOS markers, enzymes associated with energy metabolism were also evaluated. As shown in Figure 4H–I, TF significantly increased the enzyme activities of LDH and PDH in gastrocnemius. This increase was positively correlated with the NMN dose. TF was less effective in livers than in the gastrocnemius, where it significantly increased LDH and PDH activities but not significantly (Figure 4J–K). Nevertheless, TFNH significantly increases LDH and PDH enzyme activity. All doses of NMN combined with TF significantly increased the expression of IDH2 protein in gastrocnemius muscle, but only TFNH significantly increased the expression of IDH2 protein in the liver (Figure 4L–N). Muscle glycogen can also reflect the level of energy metabolism to a certain extent. It can be found that both TF and TFN can increase the accumulation of glycogen in gastrocnemius muscle fibers (Figure 4O).

### 3.5. Effect of TF and TFN on Mitochondrial Function of Mice

**Figure 5:**
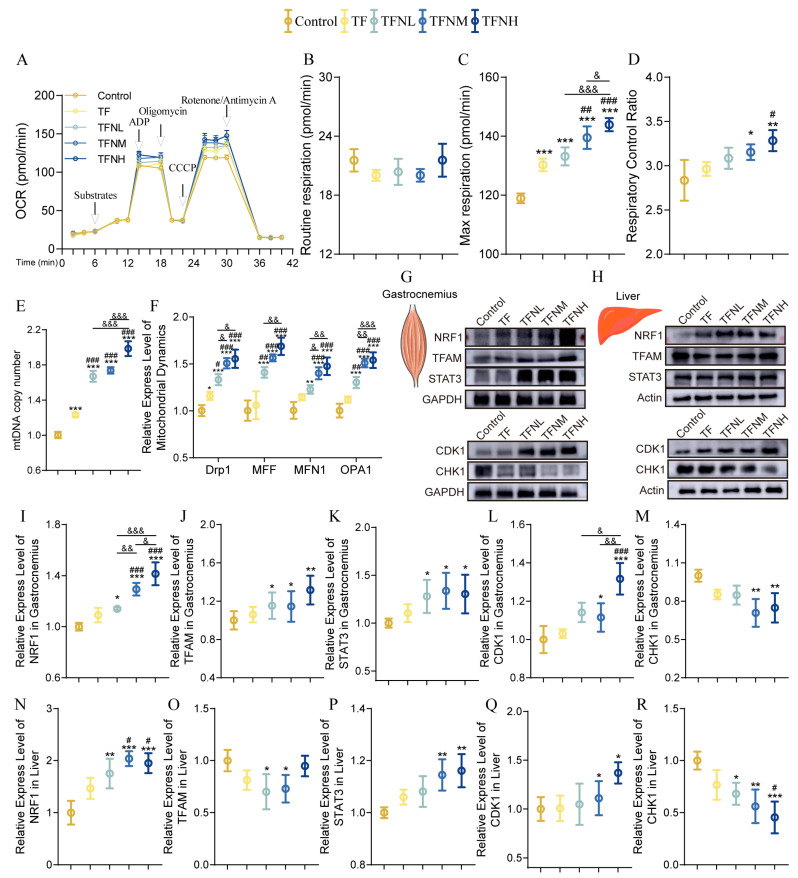
Mitochondrial function. (**A**) Mitochondrial OCR and the indicators related to respiratory function in gastrocnemius. (**B**) Routine respiration. (**C**) Max respiration. (**D**) Respiratory control rate. (**E**) The mtDNA copy number. (**F**) Relative expression of mitochondrial dynamics genes in gastrocnemius (Drp1, MFF, MFN1, OPA1). (**G**,**I**–**M**) Relative expression of mitochondrial biogenesis proteins (NRF1, TFAM, STAT3) and cell cycle proteins (CDK1, CHK1) in gastrocnemius. (**H**,**N**–**R**) Relative expression of mitochondrial biogenesis proteins (NRF1, TFAM, STAT3) and cell-cycle proteins (CDK1, CHK1) in the liver. Data were presented as means ± SDs. * represents the difference from the control group, # represents the difference from the TF group, and & represents the difference between the combined intervention groups. *, #, &: *p* < 0.05, **, ##, &&: *p* < 0.01, and ***, ###, &&&: *p* < 0.001.

Mitochondria are essential organelles responsible for energy production, and motility is directly related to mitochondrial function. Figure 5A shows the mitochondrial respiration oxygen consumption rate, and Figure 5B shows the routine respiration. As presented in Figure 5C,D, the TF group remarkably enhanced the Max respiration, and TFN significantly increased the Max respiration and respiratory control ratio (RCR) with the increased NMN level. Mitochondrial function is also related to mitochondrial dynamics and mitochondrial biogenesis. The MtDNA copy number significantly increased under the influence of TF and increased with increasing NMN dose (Figure 5E). In addition, TF promoted mitochondrial fission, which was reflected in the significantly increased Drp1 gene expression (Figure 5F). The expression levels of Drp1 and MFF genes in the TFN group were considerably higher than those in the control and TF groups. Not only that, but mitochondrial fusion was also promoted by TF and TFN. The expression levels of MFN1 and OPA1 genes were significantly increased with TF and NMN dose (Figure 5F). The expression trends of mitochondrial biogenesis-related proteins in gastrocnemius and livers differed slightly (Figure 5G–R). TF significantly increased NRF1 in the gastrocnemius and livers, had no significant effect on the gastrocnemius TFAM, but significantly reduced the liver TFAM. Compared with TF, TFN significantly increased NRF1 in gastrocnemius and livers, TFNH significantly increased TFAM expression, and restored liver TFAM reduced TF to normal levels. It is worth mentioning that TFN significantly promoted STAT3 in the gastrocnemius and livers. In addition, CDK1 protein expression was significantly increased through TFN. Correspondingly, the expression of CHK1 was reduced under the actions of TF and TFN.

### 3.6. Effect of TF and TFN on NMN Metabolism of Mice

**Figure 6:**
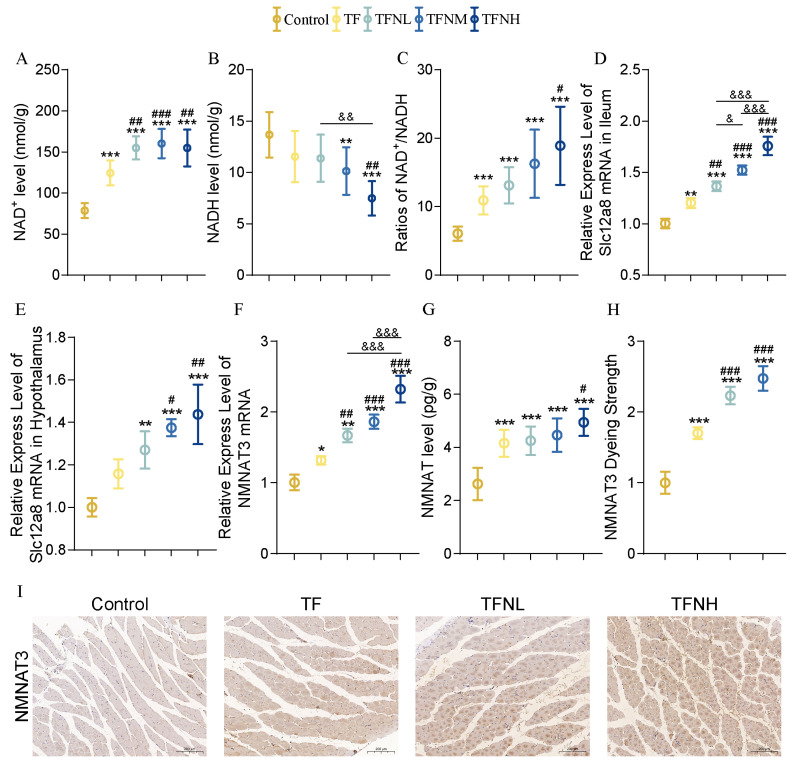
Indicators of NMN metabolism. (**A**) NAD^+^ level in the gastrocnemius. (**B**) NADH level in the gastrocnemius. (**C**) Ratios of NAD^+^/NADH in the gastrocnemius. (**D**) Relative expression of NMN absorption genes Slc12a8 in the ileum. (**E**) Relative expression of NMN absorption genes Slc12a8 in the hypothalamus. (**F**) Relative expression of NMNAT3 gene in the gastrocnemius. (**G**) NMNAT level of the gastrocnemius. (**H**,**I**) The location and relative content of NMNAT3 in the gastrocnemius. Data are presented as means ± SDs. * represents the difference from the control group, # represents the difference from the TF group, and & represents the difference between the combined intervention groups. *, #, &: *p* < 0.05, **, ##, &&: *p* < 0.01, and ***, ###, &&&: *p* < 0.001.

TF and TFN significantly increased the NAD^+^ level and NAD^+^/NADH ratios in the gastrocnemius, and the ratio in the TFNH group was considerably higher than that of the TF group (Figure 6A–C). In addition, Figure 6D–E illustrated that the expression of Slc12a8, a gene responsible for transporting NMN in the ileum and hypothalamus, was significantly increased under the promotions of TF and TFN. The expression of the NMNAT3 gene in the gastrocnemius also increased dramatically under the intervention (Figure 6F). Also, the NMNAT content in the gastrocnemius was significantly increased (Figure 6G). NMNAT gene expression and content changes were directly and positively related to the NMN dose. The tissue localization and fluorescence intensity of NMNAT3 are shown in Figure 6H–I. It could be intuitively seen that the expression of NMNAT3 in muscle tissue significantly increased with the dose of NMN.

### 3.7. Effects of TF and TFN on Gut Microbiota and SCFAs Concentrations of Mice

**Figure 7:**
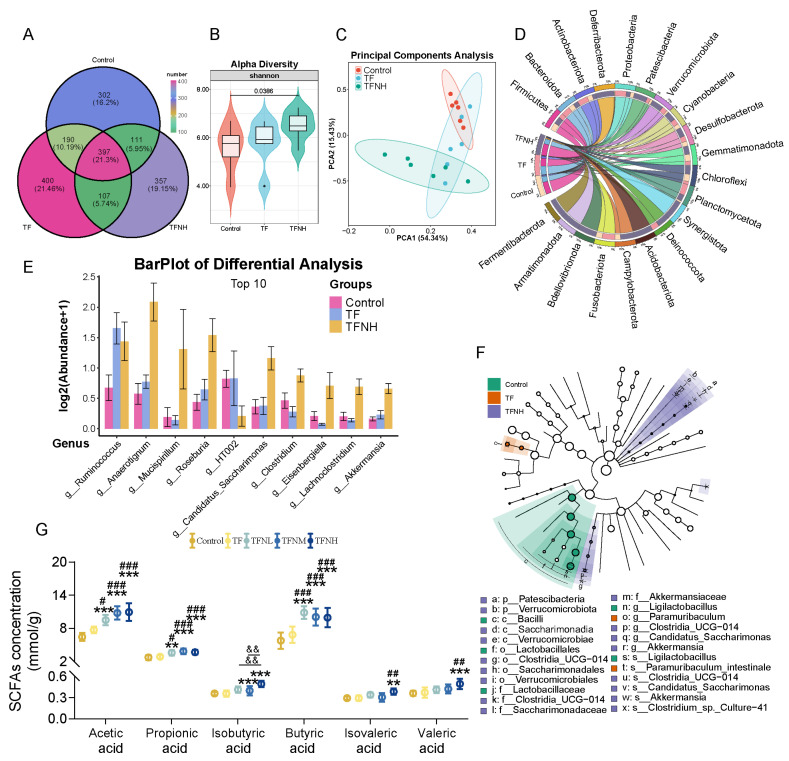
Effects of TF and TFNH on gut microbiota and the interactive analysis between gut microbiota and differential gastrocnemius metabolites. (**A**) Venn diagram. (**B**) Shannon index. (**C**) PCA method to identify group differences. (**D**) Distribution of gut microbiota at the phylum level. (**E**) The differential analysis of gut microbiota abundance at the genus level. (**F**) LEfSe analysis. (**G**) Concentration of SCFAs in cecum contents. Data were presented as means ± SDs. * represents the difference from the control group, # represents the difference from the TF group, and & represents the difference between the combined intervention groups. #: *p* < 0.05, **, ##, &&: *p* < 0.01, and ***, ###: *p* < 0.001.

16S rDNA sequencing evaluated the impacts of TF and TFN treatments on gut microbiota. 397 shared bacterial communities among groups (Figure 7A). A-diversity was markedly greater in TFNH than in the control (Figure 7B). For β-diversity, there is a notable distinction between TFNH, TF, and the control (Figure 7C). Furthermore, the main phyla observed included Firmicutes, Bacteroidetes, Actinobacteriota, Deferribacterota, and Proteobacteria (Figure 7D). At the genus level (Figure 7E), TF and TFN mice had increased relative abundance of multiple bacterial species, such as Ruminococcus, Anaerotignum, Roseburia, Candidatus_Saccharimonas, Akkermansia, and so on. Moreover, HT002 was inhabited through TFN. Next, an LEfSe analysis determined specific bacterial taxa at different taxonomic levels responsible for the most significant differences associated with control, TF, and TFNH (Figure 7F). Additionally, TFN, not TF, significantly increased the contents of multiple SCFAs, including acetate, propionate, isobutyrate, butyrate, isovaleric acid, and valeric acid (Figure 7G).

### 3.8. Effects of TF and TFN on Mitochondrial Energy Metabolism in C2C12 Cells

**Figure 8:**
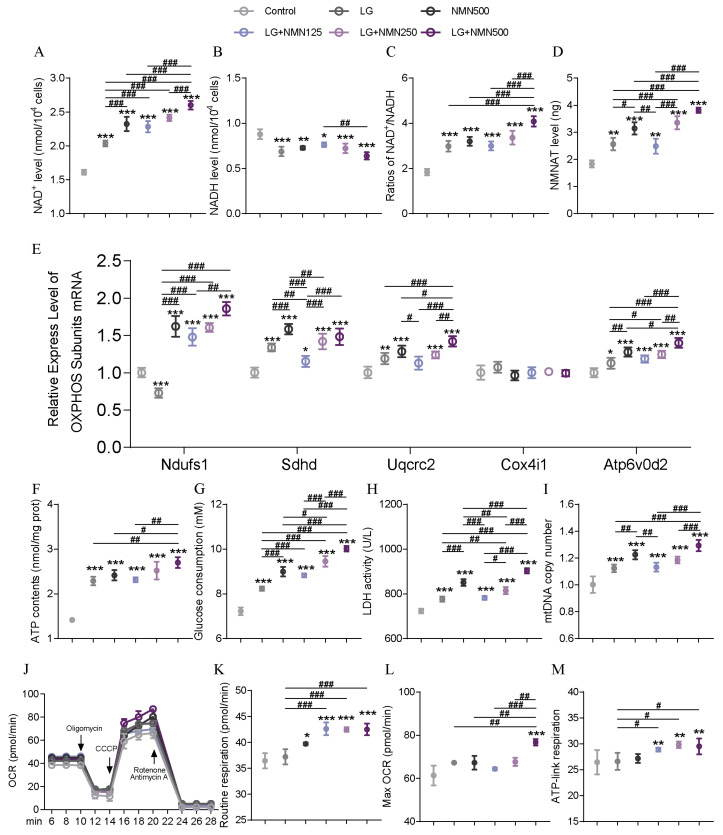
Effects of low glucose combined with NMN to simulate TF combined with NMN on C2C12 mitochondrial energy metabolism. (**A**) NAD^+^ level in C2C12 cells. (**B**) NADH level in C2C12 cells. (**C**) NAD^+^/NADH ratio in C2C12 cells. (**D**) NMNAT level in C2C12 cells. (**E**) Relative expression of OXPHOS complex subunits genes. (**F**) ATP content in C2C12 cells. (**G**) Glucose consumption in C2C12 cells. (**H**) LDH enzyme activity in C2C12 cells. (**I**) Mitochondrial DNA copy number in C2C12 cells. (**J**) Mitochondrial respiratory function in C2C12 cells. (**K**) Routine respiration in C2C12 cells. (**L**) Max OCR in C2C12 cells. (**M**) ATP-linked respiratory level in C2C12 cells. Data are presented as means ± SDs. * represents the difference from the control group, # represents the difference between the combined intervention groups. *, #: *p* < 0.05, **, ##: *p* < 0.01, and ***, ###: *p* < 0.001.

Following the in vivo observation that TF and NMN improve athletic performance by enhancing mitochondrial function, a simulated condition in vitro was achieved by treating C2C12 cells with low glucose (LG) combined with NMN. In-depth observations of mitochondrial function were conducted to verify the effects of TF combined with NMN. Figure 8 shows the effect of LG combined with NMN in C2C12 cells. Figure 8A–C show the NAD^+^, NADH levels, and the ratio in C2C12 cells. LG or NMN intervention alone significantly increased the NAD^+^ level and significantly reduced the NADH level, and the corresponding NAD^+^/NADH ratio was also significantly increased. A single intervention can considerably increase the NMNAT level (Figure 8D). After the two are combined, the effect of the single intervention on NAD^+^-related indicators is further significantly improved, showing the impact of the combined action. For OXPHOS component genes (Figure 8E), low-dose NMN combined with LG significantly increased the relative expression of Ndufs1, Sdhd, and Atp6v0d2 genes, and there was an upward trend for Uqcrc2, but it was not significant. However, combining medium-dose NMN and LG can significantly increase other genes except Cox4i1 as the dose increases. The combined intervention can play the same role as the NMN dose, which is lower than the NMN-alone intervention. For ATP content (Figure 8F), both LG and NMN can significantly increase the ATP content in C2C12 cells, and the combination of the two can further improve the ATP content. Figure 8G shows the effect of LG combined with NMN on the glucose consumption of C2C12 cells. Both LG and NMN interventions alone can significantly increase the glucose consumption of C2C12 cells, and the combined intervention can further increase glucose consumption, resulting in an NMN dose effect. Combining medium-dose NMN and LG can significantly increase glucose consumption compared with the single intervention group. For lactate dehydrogenase enzyme activity (Figure 8H), LG and NMN intervention can dramatically increase its enzyme activity. Figure 8I shows the effect of LG combined with NMN on the mitochondrial DNA copy number of C2C12 cells. Even low-dose NMN combined with NMN can significantly increase the mitochondrial DNA copy number. The high-dose NMN combined with LG showed a significantly higher increase than the LG group. Figure 8J shows the effect of LG combined with NMN on the mitochondrial respiratory function of C2C12 cells. Figure 8K shows the routine respiration of C2C12. LG did not affect the traditional respiration of mitochondria. NMN significantly increased routine respiration, and combining the two further increased routine respiration, which was considerably higher than the TF group alone. For Max OCR (Figure 8L), the combined effect of LG and high-dose NMN significantly increased Max OCR, and it was considerably higher than all other groups. For ATP-linked respiration (Figure 8M), the combination of LG and NMN significantly increased ATP-linked respiration compared to the control group. It was considerably higher than the LG intervention group alone.

## 4. Discussion

Bipedalism, followed by running, has been pivotal in human evolution [32]. Over time, the purpose of exercise has evolved from a means of acquiring food to a socially significant activity, initially associated with communication and later for entertainment purposes; its competitive nature continues to fuel ongoing interest in enhancing athletic performance. IF, a dietary regimen alternating between eating and fasting periods, has been shown to influence training adaptations by modifying nutrient utilization [33]. NMN, an effective supplement for increasing NAD^+^ levels, impacts various metabolic pathways, including energy synthesis and utilization [22]. Previous studies have investigated the combined effects of intermittent fasting with other nutritional or pharmacological interventions, such as the anti-obesity effects of IF combined with insoluble dietary fiber from okara or the anti-cancer effects of IF in conjunction with metformin [34,35]. However, the combined effects of IF and NMN on athletic performance-related parameters remained unexplored. This study used a 16:8 TF model, allowing mice to eat from 08:00 to 16:00, which marked the first instance of an in vitro test integrating TF with oral NMN supplementation at different doses. Using sports performance tests, mitochondrial-related indicators, transcriptomics, and metabolomics analyses, the effects and mechanisms of combining TF with NMN on athletic performance were identified. These findings can offer new options and a scientific basis for athletes and enthusiasts to develop personalized dietary plans and nutritional strategies.

Intermittent fasting is the most popular application of weight control, and our records also showed the same results. NMN is also an emerging intervention in the field of metabolic disorders. TF’s ability to control weight was further improved when combined with NMN. This combined effect was particularly evident in the degree of fatness, lean mass, and gastrocnemius coefficient. Since muscle quality directly influences exercise capacity, TF and TFN were considered to have the potential to enhance physical performance. Oxygen uptake capacity is vital in various oxygen-consuming sports [36]. IF could improve the aerobic exercise capacity of adults [16]. NMN could effectively increase the oxygen uptake associated with cardiopulmonary exercise [22]. After observing the increased PO_2_ and SaO_2_ levels under TF or TFN intervention, sport behavior tests were used to evaluate mice’s endurance, comprehensive strength, motor coordination, and balance ability from multiple dimensions. Even simple TF intervention could comprehensively improve the various sports performances of mice. However, the exhaustion running test showed no significant change in exhaustion distance or total work. After TFN intervention, the exercise performance of mice further improved. Given the observed increase in gastrocnemius coefficient and oxygen uptake capacity, the relevant parameters were immediately investigated further.

Humans have outstanding endurance among mammals, mainly due to their rare characteristics: abundant slow-twitch muscle fibers and sweat glands. In the preliminary experiments of this study (not yet published), it was found that, whether it was alternate-day fasting, TF, or the 5:2 diet, the gene expressions of slow-twitch muscle fibers in gastrocnemius increased. Short-term NMN intramuscular injections could reverse muscle atrophy and transform the gastrocnemius of aged mice into slow-twitch muscle fibers [21]. This experiment also further confirmed this point, especially TF under NMN supplementation conditions, which could more intuitively observe the conversion of muscle fiber types. Furthermore, Myl3, the slow skeletal muscle isoform distinct from Myh7 [37], and Mymx, which is involved in skeletal muscle development and controls the fusing of myoblasts into multinucleated muscle fibers [38], and Casq1, which initiates muscular contraction, were all upregulated through TF and TFN [39]. Slow-twitch muscle fibers are full of mitochondria, capillaries, and myoglobin, which can ensure a continuous supply of oxygen and the energy required for aerobic running. Augmenting the slow-twitch muscle fiber ratio enhanced aptitude for endurance activities [40]. This point was consistent with the observed improvement in athletic ability and changes in oxygen uptake. At the same time, the changes in the redox reaction in the body also attracted our attention.

Augmented resistance to oxidative damage is intricately linked to enhanced endurance [41]. This research demonstrated that TF effectively mitigated oxidative damage to DNA and cellular membranes during exhaustive exercise. Moreover, TFN may further alleviate oxidative damage to cellular membranes and proteins post-exercise. The correlation between oxidative damage and fatigue duration was analyzed to elucidate the possible alterations in mice’s reaction to exercise. It was found that TF and TFN may significantly enhance the resistance of mice to exercise-induced oxidative damage in three domains: DNA, cellular membranes, and proteins. In addition, after the end of the experiment, observations of gastrocnemius SOD activity, MDA content, and the GSH/GSSG ratio revealed that TF and TFN reduced the levels of oxidative stress and improved antioxidant capacities.

On the other hand, mitochondria facilitate oxidative metabolism to provide an organism with the required energy. Athletic performance is intrinsically linked to optimal energy usage and necessitates the body’s efficient energy storage and release. IF could regulate OXPHOS and TCA cycle to promote ATP synthesis [14]. NMN supplementation could also positively affect mitochondrial oxidative metabolism [42]. In this study, TF and TFN could increase the ATP contents in the gastrocnemius. This increase was most likely related to the increased OXPHOS-related indicators, such as subunit gene expression, enzyme activity, and respiratory function. Our findings were consistent with those of previous studies. For example, NMN could reduce the Ndufs4 gene inactivation or protect the mitochondrial respiratory damage caused by the CxI inhibitor rotenone, corresponding to TFN increasing the expression of the Ndufs1 gene reduced via TF [43,44]. The role of NMN in restoring electron transfer and increasing maximum oxygen consumption has also been proven [45]. Furthermore, LDH, PDH, and IDH2 additions within gastrocnemius and livers corroborated that TF and TFN augment energy metabolic activities, including glycolysis, gluconeogenesis, TCA cycle, and OXPHOS.

Mitochondria draw our attention further down. MtDNA encodes genes necessary to support mitochondrial function. IF could increase the mtDNA/nDNA ratio in mice [46]. The role of NMN metabolism in mtDNA replication has also been confirmed in mitochondrial metabolomics studies [47]. In this experiment, TF and TFN had the same beneficial effects. Incidentally, the upregulation of mtDNA may result from increased mitochondrial quality. Mitochondrial dynamics are essential for regulating metabolism, energy production, and cell differentiation. TF and NMN could positively regulate mitochondrial dynamics [48,49]. Then, increased mitochondrial fission and fusion gene expressions under TF and TFN conditions were observed. Mitochondrial biogenesis is the process of cells producing new mitochondria and is closely related to energy metabolism. After examining alterations in the expressions of the proteins associated with mitochondrial biogenesis in the gastrocnemius and livers, we posited that TF and TFN primarily govern mitochondrial biogenesis via NRF1 and STAT3. Subsequently, alterations in the cell cycle across different tissues induced via TF and TFN were observed, which seemed to correlate with enhanced muscle differentiation and exercise capability.

To ascertain the correlation between the athletic performance-enhancing effects of TF and TFN and NMN metabolism, the investigation from the standpoint of metabolites, transport, and utilization was approached. The complete enhancement of NMN metabolism via TF and TFN from the perspectives of products, genes, protein content, and location was assessed. This analysis indicated that the augmentation of mice’s athletic performance and mitochondrial energy metabolism was intricately linked to NMN.

This study also found that TFN significantly increased the richness of the intestinal microbiota of mice and increased the abundance of a variety of bacteria related to exercise capacity, such as *Ruminococcus*, *Roseburia*, and *Akkermansia*. For example, *Ruminococcus* is positively correlated with the ejection fraction and shortening fraction of the heart, and the improvement in exercise levels is inseparable from strong heart function [50]. Compared with sedentary women, women who exercise regularly have richer flora, such as *Roseburia* and *Akkermansia* [51,52]. Not only that, but *Akkermansia* in mice that exercise periodically also shows an increasing trend [53]. For SCFAs, the overall increase in short-chain fatty acid levels under TFN intervention also provides potential conditions for improving exercise capacity. SCFAs can enter mitochondria and participate in the oxidative metabolism of lactate [54]. During aerobic exercise, SCFAs can convert lactate into acetic acid through the catalytic action of LDH. Then, acetic acid enters the mitochondria and participates in the oxidative metabolism of the TCA cycle, which effectively removes lactate and supplies lactate as an energy substrate to muscle cells, providing additional ATP and delaying the onset of muscle fatigue. Acetic acid and propionic acid can promote the expression of GLUT4 protein and glucose uptake in skeletal muscle by activating AMPK, thereby increasing muscle glycogen levels [55]. They can also bind to G protein-coupled receptors to activate the expression of key enzymes such as skeletal muscle glycogen synthase and acetyl-CoA carboxylase, thereby promoting the metabolism of glucose and fatty acids. Increasing skeletal muscle energy metabolism is also inevitably related to improving athletic ability [56].

## 5. Conclusions

TF and TFN enhance mitochondrial function, improve energy metabolism, increase the abundance of intestinal microbiota and SCFA content through the NMN metabolic pathway, promote the development and transformation of gastrocnemius muscle fibers, and ultimately enhance athletic performance. These findings provide a theoretical foundation for understanding the role of TF and NMN in exercise.

## Data Availability

Data described in the manuscript will be available upon request.

## Supplementary Materials

The following supporting information can be downloaded at https://www.mdpi.com/article/10.3390/nu17091467/s1: Figure S1: Schematic diagram; Table S1: Nutrient composition of mouse feed; Table S2: Primary antibody information; Table S3: Primer sequences.

## Abbreviations

The following abbreviations are used in this manuscript:

OXPHOS	Oxidative phosphorylation
TF	Time-restricted fasting
IF	Intermittent fasting
TCA cycle	Tricarboxylic acid cycle
NAD^+^	Nicotinamide adenine dinucleotide
NMN	Nicotinamide mononucleotide
TFN	TF combined with NMN
NMNAT	Nicotinamide mononucleotide adenylyltransferase
LG	Low glucose
PCO	Protein carbonyl
MDA	Malondialdehyde
GSH	Reduced glutathione
GSSG	Oxidized glutathione
LDH	Lactate dehydrogenase
PDH	Pyruvate dehydrogenase
Cx	Mitochondrial respiratory chain complex enzymes
OCR	Oxygen consumption rate
mtDNA	Mitochondrial DNA
