# Supplementary material for: Effects of Time-Restricted Fasting–Nicotinamide Mononucleotide Combination on Exercise Capacity via Mitochondrial Activation and Gut Microbiota Modulation
Source: Nutrients. 2025 Apr 26;17(9):1467. doi: 10.3390/nu17091467 (PMC12073279; doi:10.3390/nu17091467)
Supplement: Supplementary file 1 [file nutrients-17-01467-s001.zip › nutrients-3603354-supplementary.pdf]

**Supplementary Materials**

**Table S1.** Nutrient composition of mouse feed

| **Trophic level** | | **Amino acid** | | **Vitamin** | | **Mineral substance** | |
|---|---|---|---|---|---|---|---|
| **Item** | **Content[1]** | **Composition** | **Content[1]** | **Composition** | **Content[2]** | **Composition** | **Content[2]** |
| Moisture | 90 | Lysine | 13.2 | Vitamin A[3] | 14000 | Magnesium | 2000 |
| Protein | 219 | Methionine + Cystine | 7.8 | Vitamin D[3] | 1500 | Potassium | 5000 |
| Fat | 52 | Arginine | 11 | Vitamin E[3] | 120 | Sodium | 2000 |
| Fiber | 47 | Histidine | 5.5 | Vitamin K | 5 | Iron | 120 |
| Ash | 62 | Tryptophan | 2.5 | Vitamin $B_1$ | 13 | Manganese | 75 |
| Ca | 10.4 | Phenylalanine + Tyrosine | 13 | Vitamin $B_2$ | 12 | Copper | 10 |
| P | 7.5 | Threonine | 8.8 | Niacin | 60 | Zinc | 30 |
| | | Leucine | 17.6 | Pantothenic Acid | 24 | Iodine | 0.5 |
| | | Isoleucine | 10.3 | Pyridoxine | 12 | Selenium | 0.15 |
| | | Valerine | 11.7 | Biotin | 0.2 | | |
| | | | | Folic Acid | 6 | | |
| | | | | Cyanocobalamin | 0.022 | | |
| | | | | Choline | 1250 | | |

Note: [1]. The unit is g/kg; [2]. The unit is mg/kg; [3]. The unit is g/kg IU/kg.

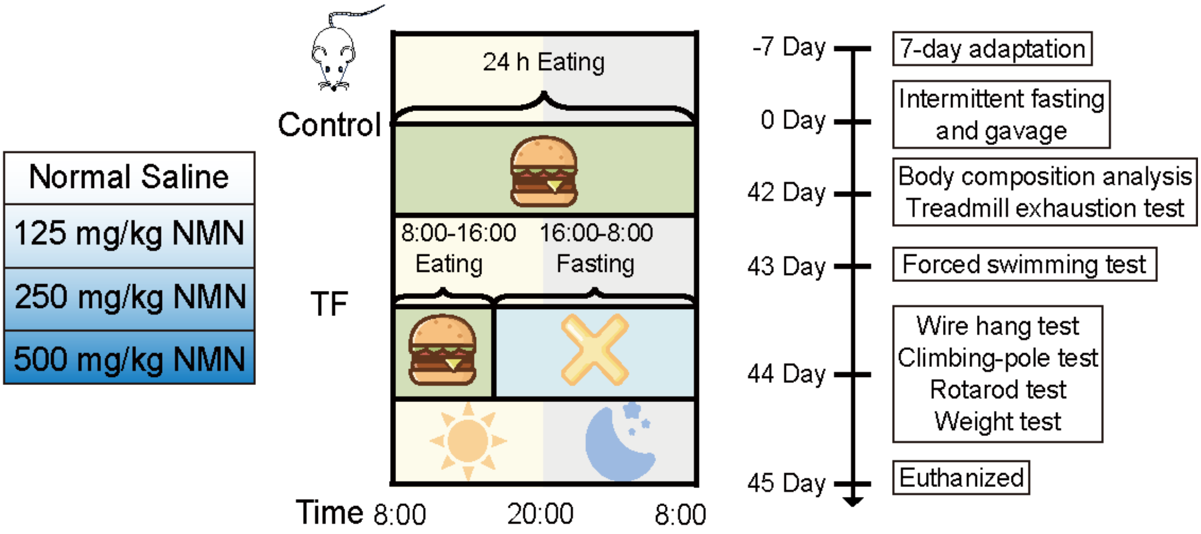


**Figure S1** Schematic diagram

**Table S2**. Primary antibody information

| Name | Brand name | Cat No | Dilution ratio |
|---|---|---|---|
| GAPDH | Proteintech | 60004 | 1:50000 |
| Actin | Abmart | M20011 | 1:1000 |
| IDH2 | Abcam | ab109588 | 1:1000 |
| TFAM | Proteintech | 22586 | 1:1000 |
| NRF1 | CST | 69432 | 1:1000 |
| STAT3 | CST | 12640 | 1:1000 |
| CDK1 | Abmart | T55176 | 1:1000 |
| CHK1 | Abmart | T55063 | 1:1000 |
| NMNAT3 | Solarbio | K109278P | 1:100 |
| MYH7 | servicebio | GB112131 | 1:200 |
| MYH1 | servicebio | GB112130 | 1:500 |

**Table S3**. Primer sequences

| | Sense sequences (5'-3') | Antisense sequences (5'-3') |
|---|---|---|
| Ndufs1 | TCACTTACAGATACGACCAT | AAGAATTGCTGCTCCATCAT |
| Sdhd | ATTTCTCCAGGACCAGCCTACC | CAGGGATTCAAGTACCCAGCAG |
| Uqcrc2 | GCTAATCCTTTGTACTGTCCT | TTGTCTCCATTCTGCTCTCT |
| Cox4i1 | TACCGCATCCAGTTTAACGA | GGGCCATACACATAGCTCT |
| Atp6v0d2 | GAACTAAGCAAAGAAGACAG | TACATACGCATAAAACACAC |
| Myh7 | CTCCTCACATCTTCTCCATC | ATTGGATGACCCTCTTAGTG |
| Myl3 | TGCCTCCAAGATTAAGATCGAG | ATCCCCACACTGCCCGTA |
| Mymx | CTGTCTGCTCTTTGTCCTCA | GCCCAATCTCTCCTTCCTCT |
| Casq1 | AACTGAAGAAGACAGCGTTT | TCGTAGGCTTTGTAATGCTC |
| Drp1 | GCGAACCTTAGAATCTGTGGA | CAGGCACAAATAAAGCAGGAC |
| MFF | CTACTCGTAGGGCTTACCAGCA | GCTCCTTCAATGGCTGCATCTAG |
| MFN1 | CCAGGTACAGATGTCACCACAG | TTGGAGAGCCGCTCATTCACCT |
| OPA1 | TCTCAGCCTTGCTGTGTCAGAC | TTCCGTCTCTAGGTTAAAGCGCG |
| Nmnat3 | AGAGACCACCTACACCAAACA | TTCACCGTTTCCATCCACTG |
| Slc12a8 | TTCCTGCTTGGTGCCGTCTGTA | ACAGGAAGCCAGGGATGAGATG |
| GAPDH | TGAACAGCACAATAAAAGAA | CCATTGAACAAGGCACCA |
| 36B4 | ACTGGTCTAGGACCCGAGAAG | TCAATGGTGCCTCTGGAGATT |
| Cytb | CCCACCCCATATTAAACCCG | GAGGTATGAAGGAAAGGTATTAGGG |
